# Young People’s Views and Experiences of a Mobile Phone Texting Intervention to Promote Safer Sex Behavior

**DOI:** 10.2196/mhealth.4302

**Published:** 2016-04-15

**Authors:** Rebecca Sophia French, Ona McCarthy, Paula Baraitser, Kaye Wellings, Julia V Bailey, Caroline Free

**Affiliations:** ^1^ London School of Hygiene & Tropical Medicine Department of Social & Environmental Health Research London United Kingdom; ^2^ London School of Hygiene & Tropical Medicine Department for Population Health London United Kingdom; ^3^ Kings College London Sexual Health Research Group London United Kingdom; ^4^ E-Health Unit Research Department of Primary Care and Population Health University College London London United Kingdom

**Keywords:** text messaging, young people, sexual health, intervention, qualitative interviews, sexual behavior, behavior change

## Abstract

**Background:**

The risk of poor sexual health, including unplanned pregnancy and sexually transmitted infections (STIs), is greatest amongst young people. Innovative and acceptable interventions to improve sexual health are required. Mobile phone text messaging (short message service, SMS) interventions have the potential to reach large numbers of people at relatively low cost, but greater understanding is needed on how these interventions should be developed and how they work.

**Objectives:**

The aim of this paper is to explore young people’s views of and experiences with a mobile phone text messaging intervention to promote safer sex behavior.

**Methods:**

We undertook qualitative interviews with young people aged 16 to 24 years as part of a pilot trial of a sexual health intervention delivered by text message in the United Kingdom. Study participants received sexual health promotion text messages based on behavior-change techniques. The message content, tailored by gender and STI status, included support for correct STI treatment and promotion of safer sex behaviors. Young people were eligible if they had received a positive chlamydia test or had more than one partner and at least one episode of unprotected sex in the last year. Telephone interviews were conducted 2 to 3 weeks after initiation of the intervention. A semi-structured topic guide was followed to explore participant experiences and a thematic analysis was conducted.

**Results:**

We conducted 16 telephone interviews with participants who had received the text intervention and an additional four interviews with those in the control group (13 women and 7 men). Intervention participants found text messages easy to understand and appearing to come from a friendly and trustworthy source. They considered the frequency and timing of messages to be appropriate, and delivery via mobile phones convenient. Receipt of support by text message allowed recipients to assimilate information at their own pace, and prompted reflection on and sharing of messages with friends, family members, and partners, thus providing opportunities for education and discussion. For some recipients, the messages had increased their knowledge of how to correctly use condoms. Some described how the messages had increased their confidence and reduced stigma, enabling them to disclose infection to a partner and/or to do so sooner and more calmly. Discussing the messages with a partner reportedly enabled some women to negotiate condom use.

**Conclusion:**

From the perspective of the recipients, the tone, frequency, and content of the text messaging-based sexual health intervention was acceptable and appropriate. Their accounts indicated that the intervention increased knowledge, confidence, and safer sex behaviors. A large-scale randomized controlled trial (RCT) is needed to assess effectiveness.

## Introduction

Globally, unsafe sex is one of the main risk factors in young people aged 10 to 24 years [1]. In Britain, young people are at greater risk of poor sexual health outcomes compared to older-age groups. Men and women aged 16 to 24 years are more likely to report at least two sexual partners in the last year with whom no condom was used [2], pregnancies in under 20 year old women are more likely to be unplanned [3], and the prevalence of chlamydia infection peaks in the late teenage years and early twenties [4]. Accordingly, most prevention efforts target men and women less than 25 years.

The Department of Health has prioritized improving sexual health in young people, yet evidence of the effectiveness of behavioral interventions using traditional methods to improve sexual health status to achieve this goal has, to date, been partial and equivocal [5,6]. Interventions that are cost-effective and broad-reaching are needed [7]. Interventions delivered by mobile phones have the potential to reach large numbers of people at relatively low-cost, and have been effective in changing some health behaviors [8]. In the United Kingdom, nine in ten young people aged between 16 to 24 years sent text messages on a daily basis to communicate with family and friends [9]. Mobile phones can provide the private, confidential, and non-judgmental support essential to a sexual health intervention targeting young people [10]. In addition, support can be delivered at any time, in any location, whenever it is needed. Behavior change techniques used in effective face-to-face interventions can be modified for delivery via text message [8].

The effects of text messaging (short message service, SMS) on key safer sex behaviors, including telling your partner about a sexually transmitted infection (STI), correctly following treatment advice, obtaining STI testing for yourself and your partner prior to unprotected sex and condom use, has not been reliably established. A systematic review of randomized controlled trials (RCTs) of mobile technology-based interventions designed to improve health or health care services identified three trials employing text messages designed to improve sexual health [11]. One trial reported increases in discussing sexual health with health professionals and improvements in STI testing and treatment; however all three trials had a high or unclear risk of bias and/or were underpowered to detect effects [12-14]. A systematic review of sexual health education delivered via social media or text message to adolescents and young adults identified five studies using text messages, three of these using a RCT design [15]. An increase in sexual health knowledge amongst those exposed to the intervention was observed in all these trials [12,16,17]. While some positive effects on behavior were observed, including increased condom use, improvement of STI screening, and a decline in multiple partners, results were not consistent across these studies. In addition, limitations included small sample size, short-term follow up, reliance on self-reported data, and poor retention.

We developed a text message-based intervention designed to promote safer sex behaviors, such as correct and consistent condom use, informing partners about STIs, and testing for STIs prior to unprotected sex with a new partner [18]. The intervention employed educational, enabling, and incentivizing behavior change functions and identified the following twelve behavior change techniques in effective face-to-face safer sex interventions: (1) information about health consequences of behavior, (2) instruction on how to carry out the behavior, (3) demonstrations of risk reduction behavior, (4) social support, (5) emotional support, (6) social rewards, (7) non-specific incentives, (8) encouragement to add objects to the environment, (9) anticipated regret, (10) problem solving, (11) action planning techniques, and (12) reframing [19-21]. Barriers to safer sex behaviors were identified from the scientific literature and functions and techniques selected to address these [21]. Messages were generated by user and expert views and refined based on feedback from potential users from eight focus groups, a survey of 100 respondents, and eight telephone interviews following receipt of the prototype messages.

The qualitative study reported here was part of a pilot RCT to establish the feasibility of a main trial to determine the effects of the intervention on safer sex behaviors. Young people aged 16-24 years were eligible if they had been diagnosed with a chlamydia infection or had more than one partner and at least one occasion of unprotected sex (ie, sex without a condom) in the last year. Participants were recruited from sexual health clinics in urban and rural areas in England. All received usual care in accordance with standards set by the British Association of Sexual Health and HIV (including treatment and support with partner notification if appropriate), and they were free to seek any other support. Participants were randomly allocated using a remote computer-generated allocation to receive either the sexual health text message intervention or the control text messages. The intervention content included messages that support correct STI treatment (taking the treatment, informing partners of the infection, abstaining from sex for one week after you and your partners are treated), and promote safer sex behaviors. Some examples of the texts are provided in Textbox 1. The messages are grounded in behavior change theory and are tailored according to gender and infection status at enrolment. The pronouns used are appropriate or the messages are gender-neutral. Some messages were only sent to some groups. For example, in focus groups women reported that they valued messages regarding how other women negotiated condom use whilst heterosexual men reported messages about how condom negotiation were not relevant to them, so they were not sent those messages. Participants who were diagnosed with an STI at enrolment were sent messages relating to correct treatment and partner notification. Participants could request further texts on a topic, for example, how to avoid condom problems or to receive tips from others. Web links to Brook (a young people’s sexual and reproductive health provider) and NHS choices (run by the National Health Service) were also provided for further information.

The frequency of messages was most intensive in the first week of the intervention, with participants receiving between 7-17 messages (varying by gender and infection status). Frequency declined over the course of the intervention and by month 12, participants received only one message per month (Multimedia Appendix 1). The control content included messages about the importance of taking part in the trial and did not include information about safer sex or behavior change. Messages were sent automatically to participants’ mobile phone.

Participants provided self-reported sexual behavioral data at baseline, 1, and 12 months. Follow-up chlamydia tests were obtained at 3 and 12 months, either via post or the designated sexual health service, whichever was most convenient to the participant.

The aim of this paper is to explore young people’s initial views and experiences of the text message intervention.

**Examples of control and intervention text messages.**

Text messages

- Intervention
  - Treatment, informing partners, and adherence
    - Most people who have an infection don’t know. Your partner(s) could be infected so it’s important to tell them that they need treatment too.
    - *"*I talked to my friend and it turned out she’d had it. And so had quite a few others I knew.*"* Text 2 to hear more.
  - Safer sex behavior support
    - Think back to a time (or times) when you had sex without a condom. Ask yourself how you could you do things differently next time.
    - If you’re new to condoms, using them can be tricky at first but it gets a lot easier with practice. Visit LINK for tips on how to use them.
- Control
  - Your participation in the texting study will help us understand more about what kind of help will improve young people's sexual health.

## Methods

### Recruitment

The study coordinator (OM) telephoned pilot trial participants who expressed willingness to participate in the interview study at enrolment. Interview participants were selected from two of the study sites, London (coded ID01) or Cambridgeshire (ID03), to ensure representation from inner-city and suburban or rural settings. Participants were purposively sampled to ensure variation according to age, gender, STI test result at enrolment, and whether they had been allocated to the intervention or control group. Control group participants were included as we wanted to explore young people’s experiences of participation in the pilot trial. Potential interviewees were given verbal and written information about the study. Informed written consent was obtained either by email or text message [22].

### Interviews

RF conducted qualitative interviews with participants by telephone 2-3 weeks after enrolment to the pilot trial. One interview was conducted by OM. Interviews took place between October 2013 and January 2014. The interviews followed a semi-structured topic guide which aimed to find out about participants’ views, experiences, and recommendations for improvements to the intervention (Multimedia Appendix 2). Questions were included on tone and frequency of text messages, views regarding the message content, any concerns about others viewing texts, what (if anything) they had learned from the texts, sexual behavior since enrolment (such as condom use and partner notification), and suggested improvements to the intervention. All interviewees were asked about experiences of being in the study, including why they agreed to participate, how they were enrolled, and their views on the randomization process and follow-up procedures (these findings are not presented in this paper). Participants were each given £20 for completing the interview. The interviews were audio-recorded and transcribed verbatim.

### Thematic Analysis

The software Nvivo 10 was used to manage data and to code transcripts thematically. After familiarization with data, RF generated an initial coding framework (with OM). RF coded all the interviews according to the framework and these were checked by and agreed with CF. Each theme was described and sub-themes identified by RF and CF. RF searched for deviant or atypical cases.

## Results

A total of 16 interviews were conducted with young people allocated to the intervention group. A further 4 interviews were conducted with young people in the control group. None of those contacted declined to be interviewed. One participant who agreed to be interviewed did not answer calls at the pre-arranged interview time. The characteristics of participants are shown in Table 1. Of the participants, 7 females (5 in the intervention group) and 4 males (2 in the intervention group) were diagnosed with a chlamydia infection at enrolment. Another male participant in the intervention group was diagnosed with gonorrhea at enrolment.

**Table 1:** Participant characteristics (N=20).

Age group		London (ID01)		Cambridgeshire (ID03)	
		Male, n	Female, n	Male, n	Female, n
16-18 years					
	Intervention	2	1	0	2
	Control	0	0	0	1
19-21 years					
	Intervention	2	3	0	2
	Control	0	0	1	1
22-24 years					
	Intervention	0	2	1	1
	Control	1	0	0	0

### Engagement With Intervention Text Messages

Most young people were positive about the intervention. The five key themes relating to user engagement with the text messages identified are (1) the importance of tone; (2) frequency and timing of texts; (3) convenience; (4) saving messages; and (5) the sharing of messages.

#### Tone

Most participants said that messages sounded as if they were coming from a friendly, trustworthy source and they liked that the messages were simple, avoided slang, and were not too long.

They didn’t use like too many big words. If it had been a load of words that I didn’t really know what they meant I’d have probably not read like the first one and then I’d have probably not read any of the others after that.

ID030012, M, 24, Intervention, chlamydia positive

Some described how it was important for them to relate to and trust the messages. They did not feel pressured, told-off, or lectured; the messages were "on their side" and enabling*.*

It was kind of like coming from a friend ‘cos it’s like it’s not speaking down to you, it’s like speaking to you. They’re like not trying to make you feel like little, they’re trying to like help you kind of thing.

ID03003, F, 19, Intervention, chlamydia positive

I didn’t feel like I was pressured into it. It was my choice if I wanted to either carry on doing the text message, if I wanted to find out the stories (links to messages on how others managed a problem or situation, see Box 1). It was very friendly, very user-friendly.

ID03002, F, 21, Intervention, chlamydia positive

One 21 year old man who had previously had genital warts felt the messages were "patronizing and dumbed-down". He said he would have preferred more statistical facts.

#### Frequency and Timing

All the participants thought the frequency of the texts, one or two a day, was just right. Their view was that they would have felt bombarded had there been any more, but less would not have been enough to reinforce the messages.

It’s been really helpful … not too much, like they don’t text too much and it gives you like information, like just little bits of information and it kind of sticks in your head so it’s been good yeah."

ID03006, F, 18, Intervention, chlamydia positive

One participant was impressed that messages continued to arrive during the weekend. It was possible to request the time of day that messages would be sent; however, none of participants had chosen to do this but felt it was an important option. Some participants who were working or at college explained that receiving texts during the day was not problematic as they tended to leave their phones in their bags and would check messages at breaks. A few mentioned the merit in sending messages out on Friday evenings when young people may be going out drinking, for example, to remind them to carry condoms before going out.

#### Convenience

Delivery via mobile phones was felt to be appropriate for young people; *kids are always on the phone.* Participants described the convenience of receiving texts on their phones as they are easily accessible and did not take up much time or attention, unlike having to go somewhere after work or trying to read through pages on the Internet. For example, "...it’s nothing like sitting on the Internet and reading all different things about it, just kind of getting some text messages every now and again" (ID03006, F, 18, Intervention, chlamydia positive). And, "‘cos it’s just a text, so even if you can’t read it right then you’d go back to read it later, it doesn’t cause any problems" (ID03013, F, 21, Intervention, chlamydia positive).

#### Saving Messages

Most intervention participants described having saved the messages they had received. Some said that they returned to the messages, prompting reflection, or kept them for future reference.

I’ve got all of them on my phone so like sometimes I’m going through my text messages… and then I go back through and read the stuff that’s come through and I do find it very helpful... but sometimes you want to go back on stuff, … if you are thinking about where your situation’s gonna be, you’re meeting a new partner and you’re like, right, we’re gonna have to have this conversation,… then you have a look and then you kind of, it helps you, it builds your confidence a bit with the tips and it’s the reassurance.

ID03002, F, 21, Intervention, chlamydia positive

The fact that the messages were not personal allayed fears connected to their discovery by other people. Phones of some participants’ were locked and could not be accessed by anyone else and some set their phones to prevent messages popping up on the screen. A couple of participants deleted their messages, one saying that he had done this as he did not want anyone else coming across them.

#### Sharing Intervention Messages

Many participants described sharing the text messages they received. For some this was done to pass on information to younger siblings or friends. One young woman had kept her messages so that she could forward them to friends if they had any problems in the future. Another young woman in the control group explained how a friend of hers, who was in the intervention group, had shared some of the messages she had received. This control participant was particularly enthusiastic about the messages and reported that she would not have been able to tell her partner about her test result had it not been for the support and tips provided through the texts. Sharing was not always intentional. One young woman said that her mother had seen the texts, and although her mother was initially angry on learning that her daughter had had a positive chlamydia test, after talking through the texts with her daughter she felt that the texts were a good idea.

An important aspect of sharing messages for some was to help to initiate a conversation, usually with a partner. For example, messages were used by some women to support or back-up negotiations on condom use.

I’ve shown him a few (messages) about condoms and that but he wasn’t listening to me and I was like oh my God … then show him the messages, yeah…he’s like well, I’m not fussed about it. I’m so like worried about it and like I know a lot about it now … I told him about that oh if I caught this again … I’d rather have a condom than catch an infection again. At first he was just like “Oh, like I really don’t like it” but after he’s seen the get pregnant or something in the future (reference to texts relating to infection and risk to infertility) which made him think as well. So I think like when you look into it deeper it helps a lot.

ID03006, F, 18, Intervention, chlamydia positive

Not all partners were as responsive:

He didn’t really pay any attention, he was just like, "Oh you’ve got one of them texts again," you know

ID03024, F, 16, Intervention, chlamydia negative

### Impact on Knowledge

Participants’ reports regarding the impact of the intervention on knowledge varied. At one end of the spectrum there were those who reported that they knew little about STIs or how to use a condom.

Well, there was this one, yeah, that said how to put a condom on, the best...(Laughs) The quick and fresh way to put a condom on... (Laughs) Because I didn’t know that much about condoms so I followed the link (to obtain further information) and I’m like, oh and it feels good when I learned how to put it on, you don’t have to use something that got oil, yeah, you don’t have to use it because the condom might burst and something like that ... oh I didn’t know that’s how you get it (chlamydia), I didn’t know, I was like, oh I need to be more careful then, I need to use a condom mostly when I meet someone new.

ID01002, F, 22, Intervention, chlamydia positive

The topics reported to be particularly helpful included how to put a condom on, how to prevent condom breakages (eg, not using oil-based lubricants), STI testing, how to talk to a partner and messages relating to building confidence, and reducing the stigma and worry about the health concerns related to the chlamydia test result.

While some of the participants reported that they already knew most of the information, they said the messages reminded them of what they had learned in the past, reinforced this information or helped them reflect on what they knew.

Most of the stuff I knew but it kind of gave me a thought, because you don’t really think about it sometimes at the time that you’re getting into anything, you just kind of do what you’re doing, but because of the texts it kind of keeps it in your mind so you know what you’re doing really before you get into anything.

ID01043, M, 18, Intervention, chlamydia negative

I think everyone should have this texting thing come out to them every day because sometimes you do forget little things that obviously you should be doing.

ID03013, F, 21, Intervention, chlamydia positive

Some participants did not find the texts helpful, describing the messages as "common sense". They tended to be older and/or negative for chlamydia at enrolment. However, they generally felt that the advice was good, but would be better targeted at those 16-18 years of age. Participants had the option to text "Stop" if they wanted to discontinue receiving the messages. One had done this and he explained that he would have preferred more "scary facts...to make kids think" (ID01042, M, 21, Intervention, chlamydia negative). One of the female participants who had sexual relationships solely with women felt that the messages on condom use and contraception, for example, were not relevant for her.

### Influence on Behavior

#### Partner Notification and Treatment

Nearly all those diagnosed with an STI in the intervention and control groups reported feeling able to notify their sexual partners of their test result. The exception was one young woman who had no contact details for a casual partner. Some of those in the intervention group said notification was done before receiving the texts, but others said that the texts about talking to your partner had helped them to have this discussion.

Participants reported lacking confidence in telling a partner about an infection and it was particularly helpful to receive messages about how others had done so. The mode of delivery was compared favorably with the approach adopted in health care settings which was described as more didactic.

When they told me first, yeah, at the hospital you have to tell him, I’m like no, I’m not going to tell him and they’re like, do you want us to call him? I’m like, no, I’m not going to give you his number and they’re like, well you have to, (laughs), you know, you have to tell him. I’m like, no, I don’t know how to, anyway, you have to, just find a way to tell him. So I wasn’t that confident ... but when I start this group (participation in the study) and they start telling me about chlamydia, that it’s not that dangerous, you can cure it, ... you have to get tested and all that so it actually helped me a lot.

D01002, F, 22, Intervention, chlamydia positive

All participants diagnosed with chlamydia said that they and their main partner had been treated and that they had not had sexual intercourse in the week following treatment.

#### Reassurance and Reduction of Stigma

The information received in text messages that chlamydia is common, that you may not know you have it, and that it is easily curable was said to have reduced concerns and stigma, which in turn increased confidence in telling a partner. Some described being distressed after receiving a positive chlamydia result and the text messages were able to give them some reassurance. They reported being able to tell partners about an infection without blaming them or being blamed.

It basically said like not to blame him kind of thing ‘cos, so ... it made it easier for me to handle the fact that I had it as well as the fact that obviously I needed to tell him so it was more comfortable like ‘cos I wasn’t like angry or whatever.

ID03003, F, 19, Intervention, chlamydia positive

The texts were also seen as helping to manage their own or their partner’s anger and re-enforce that they had "done the right thing" in telling their partner.

Well I got the text and it was like ‘Best way to tell your partner, sit down and explain it, and just say “Look, we both need to get treated”. So I did, I told him, he kicked off first of all, he weren’t very happy, and he was like ‘You’ve cheated on me’. I was like ‘No I haven’t, you blatantly know’. Then we stopped speaking for a couple of days, and then he said ‘Yeah, it’s cool, I’ve been treated’ … but yeah, that was basically it.

ID03007, F, 16, Control who had read texts of friend in the Intervention group, chlamydia positive

I probably would of (notified her partner) because even though I don’t like him and even though I think that this whole problem is caused from him … I wouldn’t want it to be passed onto anyone else, … but then this sort of study has shown me that that’s the right thing to do and really you should just tell someone.

ID03013, F, 21, Intervention, chlamydia positive

#### Condom Use and STI testing

Some young people in the intervention group said that they were now using condoms following their chlamydia test result and receiving the texts.

I’ve been with him for eight months, it’s like before I met him I didn’t use like condoms and stuff and then obviously when I found out I had chlamydia I’ve used one every single time, like ‘cos obviously I know how to put them on now... I don’t have a problem using them now, so it’s helped me in that sense as well.

ID03003, F, 19, Intervention, chlamydia positive

When asked whether their behavior change was due to their positive chlamydia result or the texts, some interviewees felt that the texts had helped improve their use of condoms. For example, in response to the interviewer question "... do you think that's more to do with the texts or was that because you got diagnosed with chlamydia?”, one said:

No, the texts, the slogans. One of the texts were … ‘use a condom’ or something like that, and I thought ‘Yes, I’m doing that!’

ID03007, F, 16, Control group – read texts of friend in the Intervention group, chlamydia positive

One woman was of the view that the text messages would not be sufficient for her to change behavior as it would be not be possible to introduce condoms if a partner refused.

My partner don’t like them so I’ve never used them before, yeah. Whenever I tell him to use them, he’s like no, you’re my wife, I’m not going to use them. You know how... (Laughs) … African men are like, no. (Laughs) … they’re like, you’re my wife so I’m not going to use it. We’re not married but that’s what he always says, oh you’re going to be my wife so there’s no point of you using them. Yeah, so and I like him, I love him, so I’m like, okay, I’m not going to use it then.

ID01002, F, 22, Intervention, chlamydia positive

Some in established relationships said they would not be using condoms with their current partner, but their intention would be to use them with any future partners and to go for chlamydia screening.

But beforehand I didn’t really think about it, I just used to go ‘yeah, that’ll be fine, it’ll never happen to me’... cos I don’t really use, well I only ever had one one-night stand anyway but, I’ve got a missus now anyway, but we don’t use a condom now, but if I did sleep with someone else now, if I split up and then see someone else I would definitely use a condom now.

ID03012, M, 24, Intervention, chlamydia positive

Some expressed the intention to go for screening check-ups and to ask their partners to do so in the future. Participants reporting behavior change were more often younger, had received a positive STI test result and/or were those not living in an inner-city setting.

### Mechanism of Action

**Figure 1:**
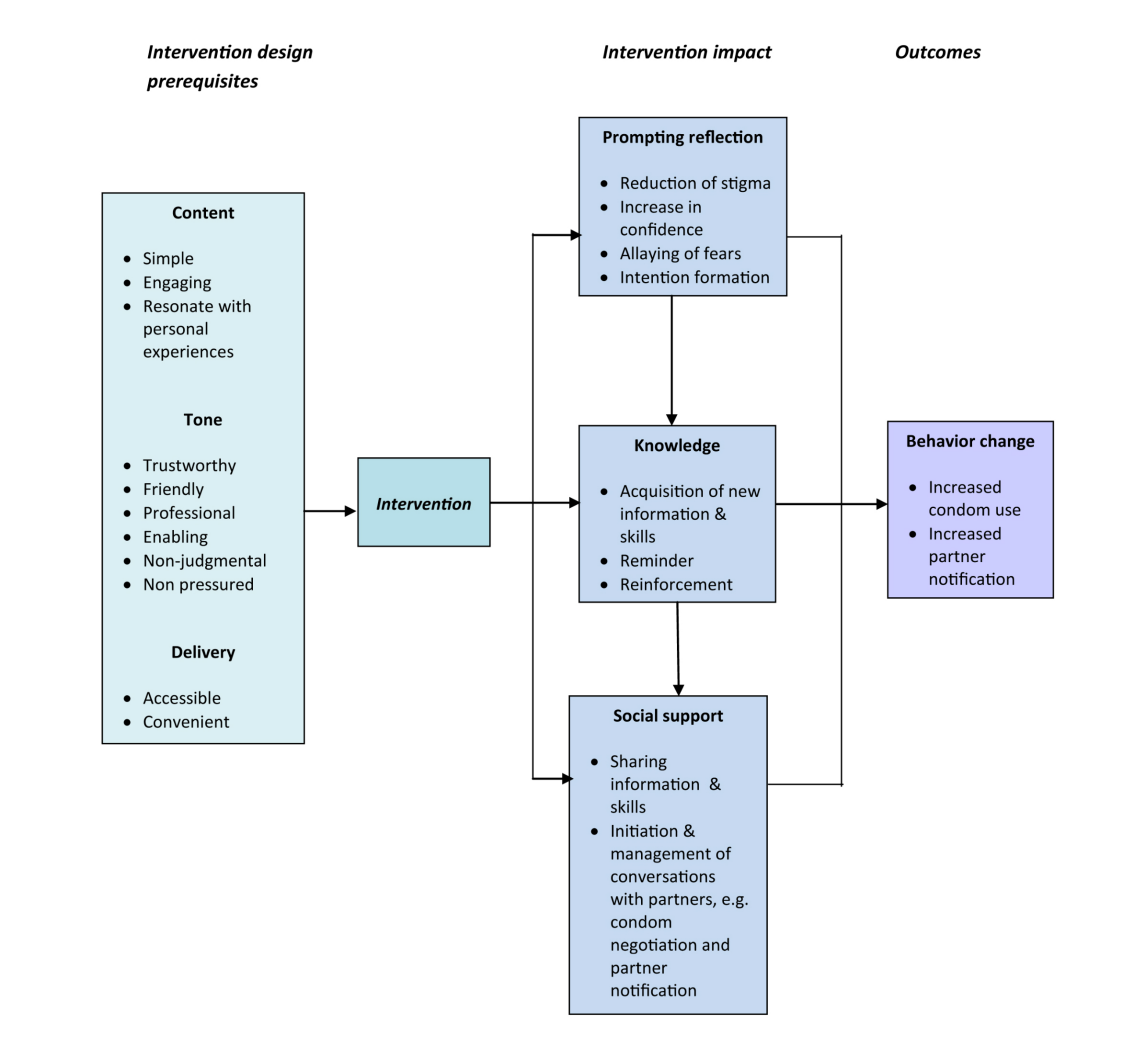
Prerequisites and mechanism of action.

Coding and analyzing the interviews led to the development of a theoretical framework for the mechanism of action, including how the intervention is hypothesized to work at increasing safer sex behaviors. The prerequisites for content and delivery and the mechanism of action are illustrated in Figure 1. The findings from the interviews suggest that the intervention could work by providing information and skills to young people via a channel that is convenient and acceptable to them. For example, the texts appeared to help by providing new knowledge on how to put a condom on or having a reminder text facilitating condom use, and breaking down assumptions about how chlamydia infection is transmitted. The messages could also work by allowing recipients to reflect on their behavior and/or help them talk to their partner about the importance of protecting themselves against STIs, such as giving them the words which could be used when having these discussions or sharing the actual texts. The fact that this was done in a way which reduced stigma and was not pressured or judgmental assisted the communication with others.

There was less suggestion from the interviews that attitudes had shifted. The one exception was in relation to stigma. Stigma associated with STIs can result in young people not accessing appropriate care and services [23], therefore its inclusion in health promotion interventions addressing sexual health is key.

## Discussion

### Principal Findings

The majority of young people we interviewed were positive about the mobile phone texting intervention to promote safer sex, particularly as it was convenient and took little of their time. The frequency, timing, and tone of the texts were appropriate for most. Receiving information and support in simple bite size messages was reported to help participants retain information. The text message medium also enabled participants to save and reflect on messages in their own time and share messages. Sharing texts provided participants with the opportunity to educate friends and siblings and acted as a prompt for discussions about sexual health and safer sex behaviors with partners. Text messages reportedly improved knowledge and confidence, and had a positive effect on safer sex behaviors, including condom use, STI testing, and notification of partners about a positive STI test result and/or hastening notification of partners.

The nature of the short content and method of delivery made the messages more acceptable than more traditional methods of health promotion such as printed leaflets. The intervention was developed using theory, evidence-based effective behavior techniques, and expert and user views. The content was designed to address attitudes, information, and behavior skills, rather than induce fear which has found to be ineffective [18,24].

### Discussion in Relation to the Existing Literature

Our findings highlighting the importance of trust, reduction of stigma, and convenience are consistent with research on what young people want from sexual health services [25]. In keeping with other research, participants viewed the mobile phone as an essential everyday item owned by most people, with easy-to-use technology [26,27]. The particular *push*, the fact that messages are sent to participants rather than retrieved by them [27], makes this a convenient, low-commitment way to receive and share information and gain support. The *always-on-you* nature of the mobile phone [28,29], enabled the mobile phone and the text messages to act as reminders and maintain the salience of sexual health behaviors. This is consistent with previous work which reported that text messages in smoking cessation acted as reminders and maintained the importance of quitting [30]. In sexual health, previous research has reported that text messages provided and reminded people of information [31]. The technology allowed participants to easily retain messages [32]. This enabled recipients to absorb information at their own pace and to refer back to messages and reflect on them in relation to their own experiences and behavior. Rereading messages has been reported in previous research where a text message-based intervention was used to support smoking cessation [30]. In this case rereading messages was used as a tool to sustain motivation for quitting rather than for reflecting on past and current behavior. Retaining messages also facilitated discussions with friends and family and partners. Retained messages were shown to partners to support participants in negotiating condom and in telling partners about being diagnosed with an STI. This is in keeping with previous research which has reported that women receiving text messages regarding contraception used this facility to retain messages to share messages and initiate conversations with partners about contraception [33]. The perception of the mobile phone as a highly personal object [32,34], combined with messages written in a non-judgmental tone may have underpinned experiences of the intervention providing support and increasing confidence. As others have shown, concern about *reputation* and perceived social expectations can inhibit communication [35], but by increasing confidence and allaying fears, our findings suggest that a text messaging intervention has the potential to provide young people with skills to overcome some of the barriers to partner notification.

The favorable reception of our text messaging intervention among the young people we interviewed resonates with findings from qualitative studies in Australia and in the United States, which have found that sexual health promotion interventions delivered via text messaging are an acceptable and convenient way to deliver potentially sensitive information and support to young people [36,37]. In accordance with our findings they found that young people favored simple messages, they reflected on the content and that they shared messages with friends. The Australian study reported no change in condom use [36].

The suggestion in our study that the text messages had a positive effect on the promotion of safer sex behaviors, particularly with reference to providing encouragement, support, and skills relating to partner notification have strong implications for infection control. Mathematic modeling suggests that the expected probability of rate of chlamydial re-infection without partner notification is 19.4%; however, if a partner receives treatment within three days after receiving treatment, this is reduced to 4.2% [38]. It is estimated that only around 40% to 60% of sexual contacts are notified by patient referral [39,40], so new strategies are needed to help improve the partner notification process. If the difficulties of notifying a partner can be lessened by giving young people the necessary skills to expedite the process, as was described in our study, and reduce time to treatment, the number of contacts informed through patient referral is likely to increase. If our trial shows the intervention is effectives it will be low cost and could be integrated with electronic systems so it is automatically sent to patients when they receive test results.

### Strengths and Limitations

The formative work done with the target group in the development of messages was key to the intervention’s acceptability. Interviews were conducted shortly after participants had received the messages in order to minimize problems with recall. Our sampling strategy ensured that there was representation of different age groups, genders, and settings, so that a broad range of views could be included.

There are challenges in conducting research by telephone, for example telephone interviewing may have resulted in more superficial and briefer responses to questions than would have been the case if the interviews had been conducted face to face [41]. However, given the nature of the intervention this was an appropriate method which allowed us to interview geographically dispersed individuals. We are reliant on young people’s self-reports which may differ from actual behavior, and we were unable to explore the extent to which any behavior change might be sustained. Young people may have provided responses that they felt would be desirable for the interviewer to hear, and they may have also felt that they needed to be positive about the intervention itself. We tried to minimize this effect by getting a member of the team (RF) who was not directly involved with recruiting participants or the day-to-day running of the project to conduct the interviews.

### Future Directions

The findings from these qualitative interviews will be used to adapt the intervention prior to an evaluation in a full scale RCT. Suggestions for improvements included refining messages so that they are relevant to lesbian, gay, bisexual, and transgender young people. Techniques could be provided to young people in established relationships on how they can negotiate behavior change without impacting on trust and intimacy. Contamination between the intervention and control groups was identified during the interviews. This could potentially lead to an under-estimate of any treatment effect. Because some participants reported sharing their text messages, we will measure this in the main trial in order to take into account any effect of contamination. However, in real-world implementation the interviews illustrate how the sharing of text messages is a good way of disseminating information and encouraging discussion. The collection of biological markers, using vaginal self-swabs for women and urine for men to test for STIs, will allow us to validate reported outcomes in a main trial. The qualitative interviews suggested the intervention may have a greater impact on the younger participants and the effect of the intervention on participants by age will also be investigated.

### Conclusion

Our research found that a mobile phone-based texting intervention was acceptable to young people and the interviews suggested it helped promote safer sex behaviors, including increased condom use and partner notification. A full scale RCT is required to establish the effects of the intervention on the acquisition of STIs.

## Appendix

Multimedia Appendix 1 Number of messages received by participants.

Multimedia Appendix 2 Topic guide.

## Abbreviations

RCT: randomized controlled trial
STI: sexually transmitted infection
