# Supplementary material for: Young People’s Views and Experiences of a Mobile Phone Texting Intervention to Promote Safer Sex Behavior
Source: JMIR Mhealth Uhealth. 2016 Apr 15;4(2):e26. doi: 10.2196/mhealth.4302 (PMC4851722; doi:10.2196/mhealth.4302)
Supplement: Multimedia Appendix 1 [file mhealth_v4i2e26_app1.pdf]

Appendix 1. Number of messages received by participants

Time line	Intervention				Control
	Women positive	Men positive	Women negative	Men negative	
	Number of messages				
Week 1	16	17	7	9	2
Week 2	8	8	8	7	
Week 3	7	6	5	4	
Week 4	6	5	5	4	
Month 2	9	9	9	9	1
Month 3	2	2	2	2	1
Month 4	2	1	2	1	1
Month 5	1	1	1	1	1
Month 6	5	5	1	1	1
Month 7	2	2	5	5	1
Month 8	1	1	2	2	1
Month 9	1	1	1	1	1
Month 10	1	1	1	1	1
Month 11	1	1	1	1	1
Month 12	1	1	1	1	1
