# Supplementary material for: Young People’s Views and Experiences of a Mobile Phone Texting Intervention to Promote Safer Sex Behavior
Source: JMIR Mhealth Uhealth. 2016 Apr 15;4(2):e26. doi: 10.2196/mhealth.4302 (PMC4851722; doi:10.2196/mhealth.4302)
Supplement: Multimedia Appendix 2 [file mhealth_v4i2e26_app2.pdf]

Texting Sexual Health Pilot
v4 Interview guide

Introduction
Thank you for agreeing to be interviewed. The purpose of this interview is to understand how being in the texting study was for you and how we can improve the texts. There are no right or wrong answers.

With your permission, this interview will be audio recorded. Everything you say will remain confidential and your interview transcript will be stored anonymously (your name can not be linked to your interview). You are free to end the interview at any time without having to give a reason. The interview could take up to an hour. We will send you £20 for completing the interview. (Check that they are able to talk for up to an hour.)

General
1. Overall what has it been like being in the study so far?

2. Why did you decide to join the study?

3. At the beginning, before you started to receive the texts, what did you think taking part would be like?

4. What was your experience of receiving the text messages?

5. How was your experience of receiving the texts different or the same to what you thought it would be like?

Intervention content
6. Did you learn anything new from the text messages? [If yes, what? if no, why not?]

7. Did you find any messages helpful? If so, which ones were they? If not, why not? [Is there a type of message that is particularly helpful e.g. advice, reassurance etc.? Is there a type of message that is less helpful?]

8. Did you save any messages to read later? [Why/why not? Which ones did you save?]

9. Did you text anything back to us? [Why/why not? What did you text?]

10. Who did you think was behind the texts (who did it seem the texts were coming from)? [e.g. did it a friend, health advisor, older sibling, doctor, researcher, academic etc.?]

11. How did the texts sound? [Were they cheesy, too embarrassing, too trusting too authoritative?]

Following the advice
12. If you followed any advice in the texts, what advice did you follow? [Did you get treated? Did you tell your partner(s) that they needed to get tested? Did you avoid unprotected sex for 1 week after you both were tested?]

13. Do you think the texts changed anything about your behaviour e.g. did they help you tell your partner, avoid sex, use condoms, etc.? [Would they have done these things anyway – i.e. if they hadn't received the texts or have done them differently]

14. Can you give me examples of when it was difficult to follow any advice given in the text messages or when the advice given was not appropriate?

Enrolment

15. How did you join the study? [Were you approached by a researcher, nurse or doctor? Was this in the clinic or over the phone?]

16. What were your first thoughts when this person told you about the study? [What was unclear? What did you understand was involved? What were you What made you decide to enrol?]

17. Did you enrol in the study over the study website or did you fill out a paper questionnaire? [How was it entering your details on the study website/filling out the paper questionnaire?]

18. What did you think about the information we asked in the questionnaire? [Was it too long/too short? How did you feel about answering the questions?]

Randomisation

19. Considering the fact that you had an equal chance of being in either group when you enrolled in the study, how much did this affect your decision to take part? Did you know what group you were randomized to? Did this affect whether or not you read the texts?]

Confidentiality

20. Were you concerned about others seeing the text messages? [If yes, how did you deal with it?]

21. Did anyone see the texts they received? If yes, was this a problem?

Frequency and timing

22. What did you think about the amount of texts you received? [Too many/too little/just right? Why?]

23. What did you think about the times you received the texts? [What times did you choose when you enrolled? What was it like receiving texts at these times?

Recommendations

24. What advice do you have about how we could improve the text message programme? [How it was delivered, content]

25. Do you have anything else to add?

Follow up interview (4 months after enrolment)

1. How has taking part in the study different/similar to what you thought it would be like?

2. Do you think the texts changed anything about your behaviour? [What did it change?]

3. What did you learn from the texts (if anything)?

4. What could you suggest about how we could make this texting program better? [e.g. make the texts more interactive]

5. If you weren't offered a voucher, would you have signed up to receive the program?

6. Do you feel any loyalty to us to stay in the trial and complete the questionnaires and provide the Chlamydia test samples? [Why/why not?]

Month 1 questionnaire

Please tell me your thoughts about completing the 1 month questionnaire

7. Did texting you before we sent the questionnaires make it more or less likely you would return it?

8. What was it like to receive the letter in the post? Were there any problems with this, i.e. confidentiality?

9. Did you complete it? Why/why not? How soon after receiving it did you complete it? Did you complete the paper version or online version?]

10. What impact did the £5 have on your decision to complete it?

Chlamydia sample

11. Did you decide to have your follow-up Chlamydia test kit sent to you at home or did you choose to attend the clinic? [Why?]

12. (For those who did home testing) What was your experience with receiving the Chlamydia test kit in the post? [Did you return the kit? Why/why not?]

13. (Woman) Did you choose to do a urine test or a vaginal swab? [Why? Did you receive sufficient information about how to do the test? Did you have any problems?]

14. Did contacting you before we sent the kits made it more or less likely you would return them? [Why/why not?]

15. What impact did the £5 sent with the kit have on your decision to provide a sample? [Why]

16. What impact did the £20 we said we'd send you if you provided a sample have on your decision to return it? [Why?]
